# Combined hysteroscopic and laparoscopic management of an isthmocele: about a case report

**DOI:** 10.1016/j.ijscr.2025.111436

**Published:** 2025-05-13

**Authors:** Aziz Slaoui, Amani Ghazalah, Meryem Lamrani, Othmane E.L. Harmouchi, Soukaina Mouiman, Aziz Baidada

**Affiliations:** Gynaecology-Obstetrics and Endoscopy Department, Maternity Souissi, University Hospital Center IBN SINA, University Mohammed V, Rabat, Morocco

**Keywords:** Cesarean scar defect, Combined laparoscopic and hysteroscopic repair, Isthmocele

## Abstract

**Background:**

Isthmocele, a cesarean scar defect, is a common complication of cesarean delivery that can cause postmenstrual spotting, pelvic pain, and infertility. Its management requires accurate diagnosis and tailored surgical approaches, especially in women desiring future fertility.

**Case presentation:**

A 29-year-old woman with a history of cesarean section presented with pelvic pain, metrorrhagia, and secondary infertility. Ultrasound and hysteroscopy confirmed an isthmocele with a residual myometrial thickness of 2.2 mm. She underwent a combined laparoscopic and hysteroscopic repair. The defect was excised and reconstructed using two layers of size 0 absorbable sutures. Postoperative recovery was uneventful, and follow-up hysteroscopy at 8 weeks showed complete resolution of the defect.

**Discussion:**

Isthmocele symptoms arise from menstrual blood retention in the defect. Deeper defects benefit from laparoscopic repair, which restores uterine wall integrity, particularly for fertility preservation. The combined approach enhances defect localization and repair precision, as demonstrated in this case.

**Conclusion:**

This case underscores the effectiveness of a combined laparoscopic and hysteroscopic approach in managing isthmocele, achieving symptom resolution and uterine integrity restoration. Individualized management and long-term follow-up are essential for optimizing outcomes.

## Highlights

• A 29-year-old woman with pelvic pain, metrorrhagia, and infertility was diagnosed with isthmocele after a cesarean section.
• The patient underwent a successful combined laparoscopic and hysteroscopic repair, with complete resolution of the defect at 8 weeks follow-up.
• The combined approach offers precise defect localization and effective repair, especially in cases aiming for fertility preservation.

## 1 Background

An isthmocele, commonly referred to as a cesarean scar defect, is characterized by a pouch-like indentation at the site of a previous cesarean section scar within the uterine wall [1]. This structural anomaly has been increasingly recognized due to the global rise in cesarean delivery rates, becoming a notable clinical challenge in gynaecology [1,2]. Patients with isthmocele may present with a range of symptoms, such as abnormal uterine bleeding, chronic pelvic pain, or secondary infertility, significantly impacting their quality of life and reproductive health. To address these symptoms, various surgical approaches have been developed, including hysteroscopic, laparoscopic, and transvaginal techniques. This article highlights the combined laparoscopic and hysteroscopic approach, illustrating its efficacy in the management of isthmocele through a detailed case report.

This case report has been reported in line with the SCARE Criteria [3].

## 2 Case presentation

We present the case of a 29-year-old woman, gravida 2 para 2, with a history of one vaginal delivery followed by a cesarean section performed three years earlier due intrauterine infection.

The patient presented with a six-month history of diffuse pelvic pain, intermittent metrorrhagia, and secondary infertility. On physical examination, her abdomen was soft with no palpable masses. Gynecological examination revealed a healthy-appearing cervix with whitish leucorrhea, and bimanual palpation indicated a tender uterus of normal size, with no evidence of lateral masses.

**Fig. 1:**
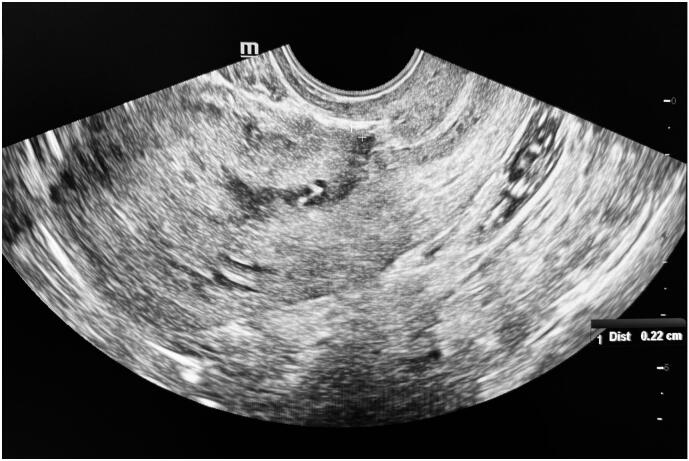
Transvaginal ultrasound revealing the isthmocele. Pelvic ultrasound shows a normal-sized uterus with a thin endometrium and a triangular, anechoic defect in the anterior uterine wall at the site of the previous cesarean section. The residual myometrial thickness at the thinnest point measures 2.2 mm (white calipers).

**Fig. 2:**
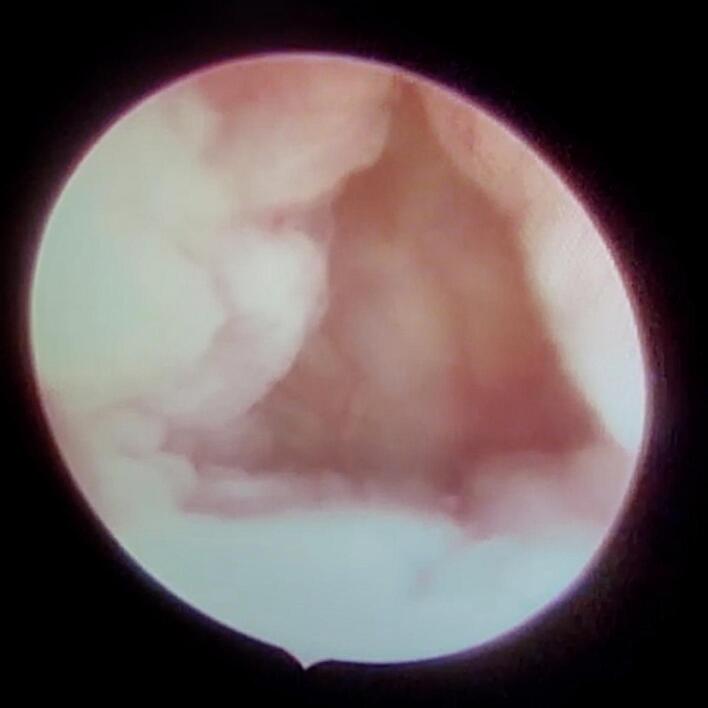
Hysteroscopic view of the isthmocele. Diagnostic hysteroscopy confirms the presence of an isthmocele localized to the upper cervical canal and the lower uterine segment. The defect is visualized as an indentation or pouch in the anterior uterine wall, covered by a thin endometrial layer.

Pelvic ultrasound revealed a normal-sized uterus with a thin endometrium and a defect in the anterior uterine wall at the site of the previous cesarean section. The defect, characterized by its triangular shape, anechoic appearance, and isthmic location, was consistent with an isthmocele. The anterior wall's residual myometrial thickness measured 2.2 mm (Fig. 1). Both ovaries appeared normal. Diagnostic hysteroscopy confirmed the presence of an isthmocele localized to the upper cervix and uterine isthmus (Fig. 2).

**Fig. 3:**
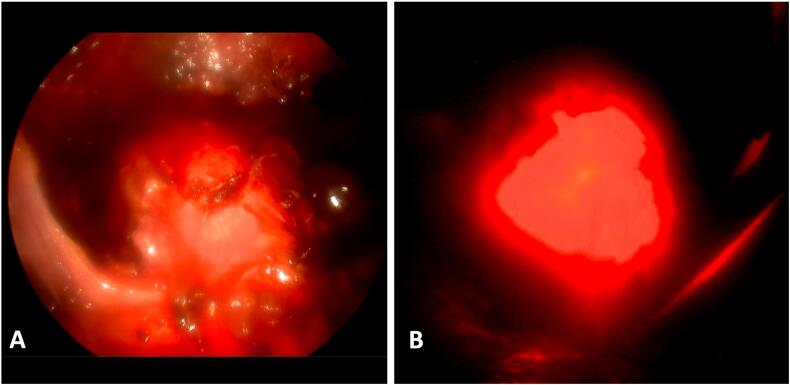
Laparoscopic visualization of the isthmocele after dissection of the vesicouterine peritoneum, assisted by hysteroscopic trans-tissular illumination. A: Laparoscopic view with the light source dimmed, showing the illuminated defect margins due to the passage of hysteroscopic light through the thin myometrial tissue. Small air bubbles are visible, corresponding to leakage of hysteroscopic distension medium through the defect. B: Laparoscopic view with the laparoscopic light source turned off, enhancing the transillumination effect and allowing precise identification of the isthmocele margins.

Following the diagnosis, a combined laparoscopic and hysteroscopic surgical approach was planned. Under general anesthesia, the procedure began with laparoscopic dissection of the utero-vesical fold to expose the anterior uterine wall. Concurrent diagnostic hysteroscopy facilitated precise localization of the defect through trans-tissular illumination achieved by dimming the laparoscopic light source. Once the defect was clearly visualized (Fig. 3), surgical repair was performed. This included excision of the defect followed by a double-layer laparoscopic closure using size 0 resorbable suture material.

The procedure was completed in 45 min with minimal blood loss (<100 mL). The patient's postoperative course was uneventful, and she was discharged on postoperative day two. Follow-up hysteroscopy performed 8 weeks later confirmed the successful repair, with complete resolution of the defect.

## 3 Discussion

Isthmocele is a recognized complication following cesarean section, with an incidence ranging from 24 % to 70 % among women with previous cesarean deliveries [4]. The pathogenesis involves incomplete healing of the uterine incision, leading to a defect that can accumulate menstrual blood, causing symptoms such as postmenstrual spotting, pelvic pain, and infertility. Infection is among the leading causes of its development after cesarean section [1]. Intrauterine infection, which was the indication for the cesarean in our patient, may be one of the etiological factors. A recent study identified several other significant etiological factors for isthmocele formation, including the number of cesarean sections, retroflexed uterus, and closure technique of the uterine incision.

Pregnancy developing within an isthmocele represents a rare but serious complication, associated with increased risks of uterine rupture, abnormal placentation (such as placenta accreta spectrum), miscarriage, and preterm birth. Early identification and appropriate management are therefore crucial, particularly in women of reproductive age [5].

Clinical presentation may vary, but there is often a triad of chronic pelvic pain, irregular menstruation and secondary infertility [1]. These symptoms arise due to the accumulation of menstrual blood within the isthmocele, leading to prolonged bleeding and irritation of the surrounding myometrium, which can cause pain. The stagnant blood within the defect may also lead to inflammation, contributing to chronic pelvic discomfort and infertility [1]. Diagnosis is straightforward using ultrasound, as demonstrated in our case, and can be confirmed by diagnostic hysteroscopy. Additionally, the role of imaging techniques such as 3D ultrasound and MRI in diagnosing and evaluating the severity of isthmocele has been highlighted in recent literature, providing better preoperative planning and postoperative assessment [6].

Management is mainly surgical. Non-invasive treatments, such as hormonal therapy and ultrasound-guided injections, are being explored as alternative or adjunctive therapies to surgical intervention [7,8]. However, these approaches require further validation through larger clinical trials [9]. Various surgical approaches have been employed to manage symptomatic isthmocele [10]. Hysteroscopic resection is effective for treating abnormal uterine bleeding but may not sufficiently restore uterine wall integrity [1]. Laparoscopic repair allows for excision of the defect and reconstruction of the myometrium, potentially offering better outcomes in terms of uterine wall strength, which is particularly important for patients desiring future fertility [1]. Transvaginal repair is another minimally invasive option that has shown promising results [10].

Recent studies have highlighted the potential benefits of combining laparoscopic and hysteroscopic procedures, particularly in cases where the isthmocele is difficult to localize laparoscopically or when concomitant intrauterine pathologies, such as polyps or adhesions, are suspected [11]. The hysteroscopic approach allows better visualization of the niche from inside the uterine cavity, guiding more accurate laparoscopic excision and suture placement. This synergy may reduce intraoperative complications, enhance the completeness of the repair, and optimize both structural and functional outcomes. The choice of surgical approach is often determined by the residual myometrial thickness overlying the isthmocele [10]. A residual myometrial thickness of <3 mm typically necessitates a laparoscopic approach, while a thicker myometrium may be amenable to hysteroscopic repair [2]. In our case, with a residual myometrial thickness of 2.2 mm, we preferred the laparoscopic approach.

In one of our previous cases, the isthmocele was not clearly identified during the laparoscopic approach, prompting us to adopt the combined hysteroscopic-laparoscopic technique for the present case. As illustrated clearly in the figures, this approach enabled precise localization and complete repair of the defect. Some authors have also suggested that hysteroscopic cauterization of the defect margins can enhance excision precision, but we did not deem it necessary in our case [11].

In our patient, the outcomes following surgical repair have been favorable, with improvements in symptoms and disappearance of the defect as shown in the hysteroscopy. Studies have also documented significant increases in residual myometrial thickness and reduction in defect size post-repair, correlating with better clinical outcomes [12]. Successful pregnancies have been reported following isthmocele repairs, indicating the feasibility of these techniques for women seeking to conceive [13]. Authors have indicated that timely intervention may prevent complications such as chronic pelvic pain and recurrent pregnancy loss [13]. Given the complexity of isthmocele and its impact on quality of life, individualized treatment plans based on patient symptoms, reproductive goals, and surgical risks are paramount. Long-term follow-up and multicenter studies are essential to establish the most effective management strategies and to refine surgical techniques.

## 4 Conclusions

This case highlights the successful management of isthmocele using a combined laparoscopic and hysteroscopic approach. Isthmocele, a recognized complication of cesarean sections, can significantly impact a patient's quality of life, presenting with symptoms such as postmenstrual spotting, pelvic pain, and secondary infertility. Accurate diagnosis through ultrasound and hysteroscopy, along with the choice of an appropriate surgical technique based on the depth of the defect, are critical for effective management.

## Author contribution

AS: study concept and design, data collection, data analysis and interpretation, writing the paper. AG: study concept, data collection, data analysis, writing the paper. ML: study concept, data collection, data analysis, writing the paper. OEH: study concept, data collection, data analysis, writing the paper. SM: study design, data collection, data interpretation, writing the paper. AB: study design, data collection, data interpretation, writing the paper.

## Consent

Written informed consent was obtained from the patient for publication of this case report and any accompanying images. A copy of the written consent is available for review by the Editor-in-Chief of this journal.

## Ethical approval

Ethical clearance was not required for this case report as it involved only a single patient and did not involve any experimental or invasive procedures. Our institution name is University Mohammed V of Rabat.

## Guarantor

The corresponding author is the guarantor of submission.

## Research registration number

Not applicable.

## Provenance and peer review

Not commissioned, externally peer-reviewed.

## Funding

There are no funding sources to be declared.

## Conflict of interest statement

The authors declare that they have no competing interests.

## Data Availability

Supporting material is available if further analysis is needed.
